# A Capacitive Touch Screen Sensor for Detection of Urinary Tract Infections in Portable Biomedical Devices

**DOI:** 10.3390/s140813851

**Published:** 2014-07-30

**Authors:** Carlos Honrado, Tao Dong

**Affiliations:** Department of Micro and Nano Systems Technology, Faculty of Technology and Maritime Sciences, Buskerud and Vestfold University College, Postboks 2242, N-3103 Kongsberg, Norway; E-Mail: carlos_honrado@hotmail.com

**Keywords:** touch screen sensor, urinary tract infection, capacitance detection, portable biomedical devices

## Abstract

Incidence of urinary tract infections (UTIs) is the second highest among all infections; thus, there is a high demand for bacteriuria detection. *Escherichia coli* are the main cause of UTIs, with microscopy methods and urine culture being the detection standard of these bacteria. However, the urine sampling and analysis required for these methods can be both time-consuming and complex. This work proposes a capacitive touch screen sensor (CTSS) concept as feasible alternative for a portable UTI detection device. Finite element method (FEM) simulations were conducted with a CTSS model. An exponential response of the model to increasing amounts of *E. coli* and liquid samples was observed. A measurable capacitance change due to *E. coli* presence and a tangible difference in the response given to urine and water samples were also detected. Preliminary experimental studies were also conducted on a commercial CTSS using liquid solutions with increasing amounts of dissolved ions. The CTSS was capable of distinguishing different volumes of liquids, also giving an exponential response. Furthermore, the CTSS gave higher responses to solutions with a superior amount of ions. Urine samples gave the top response among tested liquids. Thus, the CTSS showed the capability to differentiate solutions by their ionic content.

## 1. Introduction

**Figure 1.**
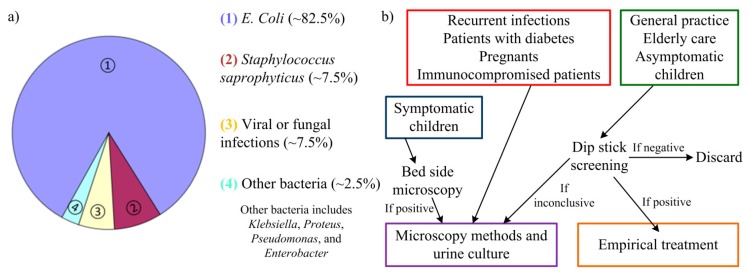
(**a**) Prevalence of the main uropathogens; (**b**) Guidelines used in common preliminary detection methods of urinary tract infection (UTI) using dip stick screening and bed side microscopy. Figure adapted from [4].

Incidence of urinary tract infections (UTIs) is only second in frequency when compared to incidence of upper respiratory infections. Nonetheless, bacteriuria detection (a common procedure for UTI detection) is the most requested clinical procedure with large laboratories examining 200–300 urine samples each day. Whereas most upper respiratory infections have a viral etiology (and so require little to none medical intervention), UTIs are mainly caused by enteric bacteria. From these, the most common organism is *Escherichia coli* (*E. coli*) with 80%–85% of the cases originating from these bacteria. *Staphylococcus saprophyticus* are other bacteria responsible for 5%–10% of cases of UTI [1]. In some rare cases, UTIs can be caused by viral or fungal infections [2]. Other groups of bacteria as *Klebsiella*, *Proteus*, *Pseudomonas*, and *Enterobacter* can also cause UTI but are typically related to abnormalities of the urinary system or urinary catheterization [3]. Prevalence of these urinary tract pathogens (uropathogens) is depicted in Figure 1a. Antimicrobial therapy is then needed to eliminate the infecting organisms and an early detection of the infection can help prevent more severe sequelae [4,5].

However, the current approaches to detect UTI are usually slow and require specific equipment. The traditional basis for uropathogens detection relies in urine culture, which involves laboratory testing (such as blood agar, for instance). Several days may pass from the urine sampling before a definite diagnosis is possible. The approaches used for UTI detection can comprise microscopic methods [4,5], using Gram's method [6]; enzymatic methods, as catalase test, glucose oxidase or reagent strip testing (dip stick screening) [7]; colorimetric filtration [8]; bioluminescence reactions [9]; photometry detection of growth [6]; electrochemical methods [10]; turbidimetric screening [11]; limulus amoebocyte lysate endotoxin test [12]; or the Malthus system [13]. For these methods, urine culture from the patient's urine sample is usually necessary to detect and quantify the bacterial growth. From the aforementioned approaches, microscopic methods are the current “golden-standard”, usually requiring a previous cell culture. Still, there are quick, preliminary methods used to screen fresh urine for UTI detection before recurrence to these methods. The guideline used for these methods is present in Figure 1b.

The urine sampling process for bacteriuria detection can also become a time-consuming and frustrating task due to some patients' inability to cooperate (e.g., the elderly and babies). Despite being a common sampling process, the act of collecting a urine sample from midstream can be extremely difficult in the patient groups mentioned. Sampling methods as catheterization, suprapubic aspiration or sterile adhesive pads for absorption can be used, but all present severe drawbacks, mainly pain and discomfort in the patient [4,14–17]. A possible solution could be using common disposable diapers as the container of fresh urine samples, used afterward for the UTI detection [4]. A portable biomedical device could then be used to perform a quick analysis of said urine sample, giving an *in situ* result for the UTI detection and avoiding recurrence to a laboratory.

A possible way to bypass laboratory testing and more complex detection methods can be based on the urine composition of a UTI patient. In a normal situation organic molecules and ions can be found dissolved in urine, which is a solution mainly comprised by water (approximately 95%). The variability of urine composition can be high, varying between patients and also over time. Organic molecules as urea, creatinine, uric acid, carbohydrates, enzymes and fatty acids, for instance, can be encountered. As for the ions the most common are sodium (Na^+^), potassium (K^+^), chloride (Cl^−^), magnesium (Mg^2+^), calcium (Ca^2+^), ammonium (NH_4_^+^), sulphates (SO_4_^2−^) or Phospates (H_2_PO_4_^−^ and HPO_4_^2−^, for instance) [18]. Bacteria are not usually part of urine (as seen from the presented list) nor are most of their metabolites. The presence of nitrite (NO_2_^−^), for instance, can signal the presence of relevant *E. coli* colonies as it is one of the main metabolite of these enteric bacteria [19,20]. A specific method to detect these bacteria and metabolites could then perform a viable UTI detection. For this sort of detection the variability within different urine samples must be taken in consideration so a specific sampling protocol for homogenization of the urine conditions must be developed in the future.

Won and Park [21] recently reported a DNA detector with the potential to become a feasible biomolecular detection platform. This detector relied on a touch screen, which skipped any sample preparation phase by simply placing the samples directly on the modified surface electrodes. DNA concentrations down to 9.2 × 10^−4^ ng/μL were detected by the touch screen proving the potential behind this technology for biological sensing. Given the results shown from this device, implementation of a touch screen based UTI detection device can prove to be a highly sensitive tool. Also, by using such a mainstream technology as touch screens, commercial solutions are already available to use in research. With their hand-size and easy-to-use characteristics, future portable biomedical devices can be developed based on touch screens.

The presented work is based on this premise and follows the concept already proposed in [22]. Herein, we study the application of a capacitive touch screen sensor (CTSS) as a feasible UTI detection system. Finite element method (FEM) simulations were firstly conducted to assess the theoretical behavior of the touch screen model to *E. coli* and two liquid samples (urine and water). Experimental testing then expanded this study with the preliminary tests assessing the response of the capacitive touch screen to different liquid solutions: DI water; liquid water; salt water and a fresh urine samples. Understanding the response of the sensor to liquids and different types of solutions is of major importance in the development of a functional UTI touch screen based detector.

## 2. Working Principle of the CTSS

As proved in [21], a touch screen can prove to be a valuable tool for biomolecular detection, holding the potential to perform high-sensitive bioassays. Touch screens have been increasingly used in so-called “smart-phones”, detecting and localizing a touch-event in a specific display area [23]. There are several types of technology used to detect the touch-events, as infrared, surface acoustic wave (SAW), resistive detection or capacitive detection, being the last the current standard in terms of smart-phones [24]. Capacitive touch screens can, however, present two different technology concepts and interaction between touch panel, controllers and the touch-event [25,26]. One approach uses the self-capacitance method (or surface capacitive response), where a small electrical current (20–500 μA) is created by the coupling of the touch-event creator (say, in the most common example, a human finger) to the touch panel surface. The controller detects this induced current, calculating the distance between that touch-event and the electrodes, distributed in the corners of the touch panel. Only a single touch-event can then be detected by the touch screen at each time, limiting their application. In contrast, the mutual-capacitance method (or projected capacitive response) allows the detection of multiple touch-events at the same time. This is accomplished by sensing capacitive changes on the electrodes, patterned on the touch panel, caused by the interaction with the human finger. Since each interaction will have a specific electrode combination and signal strength, different touch-events can be then localized and distinguished.

**Figure 2.**
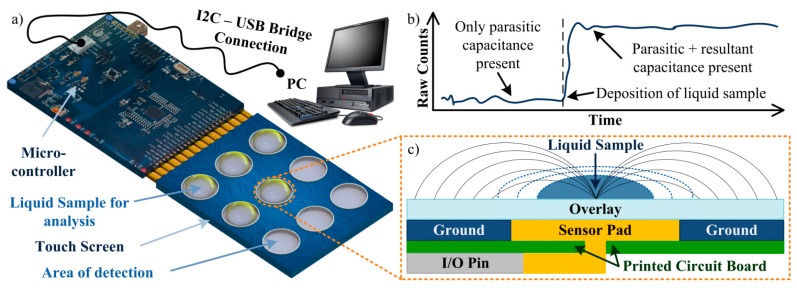
(**a**) Schematic representation of the system used. A micro-controller is connected to the touch screen used. The different areas of detection are represented, showing how a liquid sample is allocated and analyzed. An I2C – USB Bridge is also used to connect the micro-controller to a personal computer (PC), allowing the retrieval of experimental data; (**b**) Expected results from the touch screen algorithm. By deposition of a liquid sample on top of the sensor pad a change on the capacitance will lead to an increase on the RCs; (**c**) Structure of the touch screen, with a self-capacitance equivalent model. An electric field (solid lines) is created between the sensor pad and the surrounding ground, passing through the overlay. An I/O pin controls the sensor pad, which, together with ground and overlay are set onto a printed circuit board (PCB). The liquid sample is allocated on the center of the sensor pad with varying volume (dashed lines).

Throughout the presented work, a self-capacitance equivalent model is used to describe the implemented touch screen (which structure is presented in Figure 2c) [25,26]. In this system, the touch sensor constantly measures a baseline capacitance value (*C_X_*) which can vary according to the environmental conditions. If no material is in contact with the touch screen, a parasitic capacitance (*C_P_*) is measured, *i.e*., *C_X_* = *C_P_*. This capacitance takes in consideration all the effects from the touch screen parts, as the interactions between sensor pad, overlay and ground or the pin capacitance from the controller, for example. Thus, *C_P_* represents the intricate electric field created by all the components. If, however, a new material (such as the human finger) creates a touch-event, a new parallel capacitor will be created between the sensor pad and the material through the overlay. This resultant capacitance (*C_R_*) can then be defined by

(1) $$C_{R}=\frac{\varepsilon_{0}\varepsilon_{r}A}{D}$$

where *ε_0_* is the free space permittivity; *ε_r_* the relative permittivity associated with the new material; *A* the area of overlap between the added material and the sensor pad; and *D* the overlay thickness. As consequence, *C_X_* will now be equal to

(2) $$C_{X}=C_{P}+C_{R}$$

This difference between the initial state (given only by *C_P_*) and the new state (given by the sum of *C_P_* and *C_R_*) will be used in this work to understand the interaction between the touch sensor and the various solutions tested. The algorithm used by the touch sensor represents the sensor capacitance in terms of a digital count, or Raw Count (RC). As the numerical value of capacitance increases, so does the RC value increases and, depending on the strength of the signal, a touch-event can be detected by the sensor. Response in this case can be broadly defined as the measure of how much the output (RC) change for a given change in the input (material in contact with the sensor). An expected result from the algorithm is presented in Figure 2b. The overall system used in this work is represented in Figure 2a.

## 3. Modeling and Simulation of a CTSS

Before the realization of preliminary experimental tests, some theoretical tests of the CTSS concept model to implement should be performed. For these tests a FEM simulation is suitable as it allows control over the model created and feasible results as predictions of the real model behavior. For the 3D simulations of the capacitive touch screen the ACDC module from the commercial software COMSOL Multiphysics^®^ was used. The model was considered to have charge conservation and zero charge. The structure of the model mimics the one from a single button on the capacitive touch screen used in the experiments (Figure 2c). The PCB substrate was defined as a squared layer with 10 mm of side and 1 mm of thickness with part of its top being defined as the ground of the simulation. A circular area (radium = 2.5 mm) in the center of this layer was defined to be the terminal (1 V), thus creating the sensor pad. On top of the substrate a polypropylene (PP) cover foil, working as the overlay, was allocated for insulation and protection purposes, presenting a thickness of 70 μm. Also, an air region was defined surrounding the model in order to allow propagation of the generated electric field.

**Figure 3.**
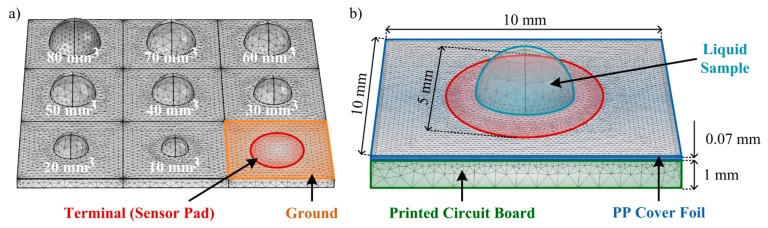
(**a**) Definition of the module parameters in the model with the PCB substrate being defined as the Ground and the centered circular area (Sensor Pad) being defined as the Terminal. The array demonstrates some the different volumes of solution (urine or water) tested, from 10 mm^3^ to 80 mm^3^; (**b**) Representation of the simulation model implemented for the self-capacitance equivalent touch screen with its main dimensions. Layered structure of the model, with a PCB substrate and a PP Cover Foil with a Liquid Sample located on top.

The conducted simulations tested the response of the touch sensor to different amounts of *E.coli*, with a relative permittivity (*ε*) of 100 [27]; urine, presenting a *ε* = 76.1 [28]; and water, with a *ε* = 80, well-known in literature. A semi-sphere, representing the liquid drop under test, was defined on the center of the model (aligned with the sensor pad) and its volume was changed according to the amounts under test. For the *E. coli* testing, some considerations were taken based on the studies already presented in [29]. UTI urine has a minimum concentration of 10^5^ colony forming units/mL and each single bacterium, part of these future colonies, has a maximum volume of 1.1 μm^3^. Considering that a 1 mL sample adheres to the touch sensor surface (by an immunoassay, for instance), all the bacteria present (before the colony-formation) would be fixed in the pad area and a total volume of bacteria of 1.1 × 10^5^ μm^3^ would then be present. As a simplification, a sphere with 30 μm of radius could then represent this volume as it is simplest shape that can be coherently simulated and best represents the shape of the bacteria. In the simulations, radiuses from 60 to 600 μm were tested, representing the *E. coli* in amounts of UTI urine from 2 to 20 mL, respectively. As for the other liquids, the volumes of solution tested varied from 10 mm^3^ (10 μL) to 100 mm^3^ (100 μL), with an increment of 10 mm^3^. In this model the contact angles between the liquids and the surface were neglected for simplification. The simulation model is depicted in Figure 3.

## 4. Experimental Setup for the CTSS Testing

For the experimental tests the microcontroller CY3280-20x66 Universal CapSense Controller and the touch screen CY3280-BSM Simple Button Module (bought from Cypress Semiconductor Corporation, San Jose, CA, USA) were used. After installation of the enclosed software, both components were ready to be programmed and used. Following the manufacturer instructions, the CY3280-20x66 Universal CapSense Controller was programmed to give a real-time RC reading from a specific button. Regarding the CY3280-BSM Simple Button Module, simple tests confirmed the correct programming of the touch screen, as pressing each button with a finger and observing the correspondent LED light-up.

As for the tested solutions, regular tap water and DI water were utilized. A salt water solution was also prepared by adding 3.5 g of NaCl to 100 mL of tap water, thus obtaining a solution of NaCl with a concentration of 35 g/L. The urine samples were obtained from fresh post-night urine, collected in the midstream, and properly stored in sterile containers. These samples were kept in a controlled environment and tested within 2 h after the collection.

To perform the tests, samples of each solution were dropped onto the touch sensor surface, in different buttons, with a pipette. The amount of sample was varied from 10 μL (10 mm^3^) to 100 μL (100 mm^3^), with an increment of 10 μL. Each amount of solution was tested five times. In order to protect the electronic components of the touch screen, a PP cover foil (bought from Biovendis, Mannheim, Germany) was attached to the surface. Initial tests proved that the addition of this layer to the touch sensor did not change the final results. Each test performed was conducted singularly, with the touch screen only measuring the RCs for the specific button in case. The RC value given by the touch sensor was registered before and after the deposition of any sample (due to small changes in the RC baseline value of each button). A Raw Count Difference (RCD) for each test was then calculated. After the realization of nine tests (corresponding to the nine used buttons), the touch sensor was cleaned using an ethanol solution, preventing adhesion of unwanted particles and remnants of previous tests (especially in the case of urine samples).

## 5. Results and Discussion

**Figure 4.**
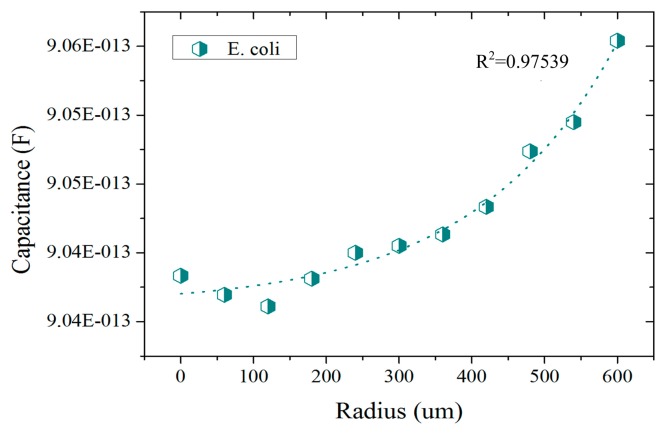
Capacitance readings of the simulation model for *E. coli* detection with increasing radius of bacteria colonies.

The presented results correspond to the preliminary studies conducted in the development of a CTSS based UTI detector. In these studies the response of the CTSS to different liquid solutions was assessed. In the FEM simulations conducted, a first set of studies was conducted to better understand the sensitivity of the sensor to *E. coli* bacteria. Some studies were already conducted in [29] but a projected capacitance method was used in the referred case. Here the self-capacitance equivalent model was used instead and the results are presented in Figure 4. In these, an exponential response from the CTSS to increasing amounts of *E. coli* is visible. This confirms the capability of the sensor to distinguish different amounts of bacteria upon its surface. Furthermore, the difference between the response for no bacteria present (radius = 0 μm) to superior amounts of *E. coli* (radius > 400 μm) was in the fF order. Despite being a small capacitance change, there are already some chips capable of detecting alterations in the capacitance of this order [30]. If integrated in a CTSS system, these chips could then detect and quantify the presence of *E. coli* in a urine sample through the capacitance change.

**Figure 5.**
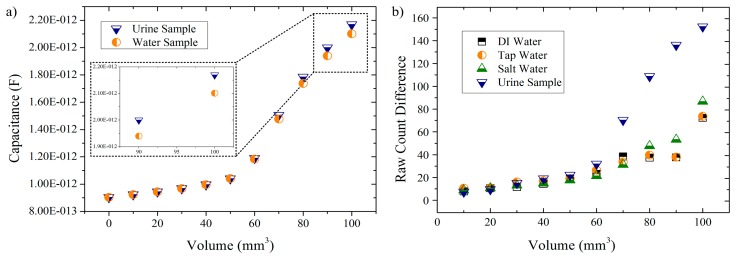
(**a**) Capacitance readings of the simulation model with increasing volumes of liquid sample. The inset portrays the last two measured values; (**b**) Raw Count Difference variation with increasing volumes of solution used. Each value is the average of five tests performed for each volume per solution.

In the second set of simulations, two different solutions (urine and water) were tested and the capacitance readings of the model were registered (Figure 5a). Regarding the experimental tests, they were aimed to not only use the same solutions in the simulations (for comparison) but to add two more. The idea was to start from a deionized solution and gradually increase the amount of dissolved ions in the solution in order to analyze how the CTSS would respond to this rise of ionic compounds. Given that nitrate ions are the main metabolite of *E. coli*, an increased population of these bacteria would result in increase of the ionic content of the urine sample. A future portable device could then perform UTI detection based on ionic properties of this sample. Therefore, the liquid samples under study were DI water, tap water, salt water (tap water with a NaCl concentration of 35 g/L) and fresh urine samples. The results obtained for these solutions are presented in Figure 5b.

In the simulation results (Figure 5a) it is possible to see an exponential response of the model to increasing volumes of liquid samples. For both solutions, an exponential growth is observed with a critical increase in the response given being observed for volumes greater than 60 mm^3^. Also, a similar response by the touch sensor model to both tested solutions is observed for lower volumes whereas for greater amounts (>60 mm^3^) differentiated responses are obtained (as seen in the inset for volumes of 90 mm^3^ and 100 mm^3^). This difference in responses is, for the volumes presented in the inset, in the order of the 0.07 pF, which is a rather small value when compared to the capacitance readings obtained. Still, this small difference can be availed in practical terms to differentiate the solutions tested.

Concerning the experimental results (Figure 5b) it is also possible to observe that, regardless of the solution under analysis, the RCD value is exponentially increasing as the volume of solution used also increases. This not only is in accordance to the simulation results as it also demonstrates that the CTSS is capable of distinguish different amounts of a liquid placed upon its buttons. Also in line with simulation results is the response given by the CTSS to lower amounts of solution (≤60 μL) as negligible differences in the RCD between the different solutions are observed. Only for superior values it is, generally, possible to observe differences between each solution. Exceptions are DI water and tap water solutions, which proved to always have a similar response by the sensor, even as the amount of solution used increases. The salt water solution presented higher responses by the CTSS when compared to the other two water-solutions with small differences being observed though. In the case of the urine sample, it is possible to observe a relevant difference from the remaining solutions as early as 70 μL, with a major increase in the RCD happening onwards. This sudden increase in the RCD can be related to a better coupling between the sample and the CTSS surface due to the increased volume of liquid deposited. The same behavior was observed in the simulation results. A correlation between the small capacitance differences measured in the simulation and the obtained RCDs can then be made, with the CTSS proving to be able to detect these minor changes in capacitance and give a definite differentiated response. Furthermore, these results also prove that the CTSS gives a greater response to solutions with a superior amount of ions, being capable of distinguish between solutions by their ionic content (especially urine samples).

## 6. Conclusions and Future Work

The high incidence of UTI, when compared with other infections, leads to a high request for bacteriuria and, thus, to an increasing need of viable urine samples. With the retrieval of these samples being a time-consuming and frustrating task, disposable diapers can be availed to more easily retrieve these samples. A portable biomedical device, based on a CTSS, could be used afterwards for UTI detection *in situ*.

The preliminary studies on a CTSS based UTI detector were presented. FEM simulation results showed an exponential response of the touch sensor to the increasing amounts of *E. coli* and liquid samples. In the first simulation set, the touch sensor presented differences in the fF order after the addition of the *E. coli* to the model which can be practically detected despite small. As for the second set of simulations, a definite difference in the response given by the CTSS to urine and water samples was observed. In experimental testing, the response of the CTSS to different liquid solutions, with an increasing amount of dissolved ions, was assessed. DI water, liquid water, salt water and fresh urine samples were the solutions tested. Results show the CTSS distinguishing different amounts of liquids and giving an exponential response as those amounts increased. These results are in accordance with simulation results. Moreover, greater responses were observed for solutions with a superior amount of ions, with the urine samples attaining higher RCDs than the remaining solutions. This proves the capability of the CTSS to distinguish solutions by their ionic content.

**Figure 6.**
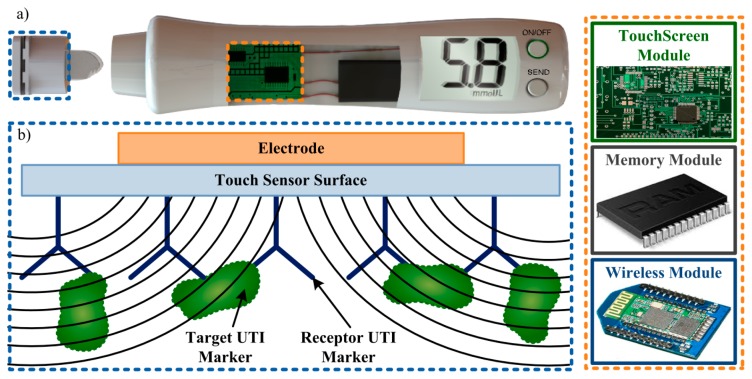
(**a**) Conceptual design of a portable device for UTI detection with its main modules (at the right side). The TouchScreen Module performs the capacitance detection and calculates the result, giving a response that is stored in the Memory Module. The stored data is then transmitted, by the Wireless Module, to a central computer and/or smart-phone where the results can be analyzed and interpreted by the responsible physician; (**b**) Working principle of a possible touch screen based solution for UTI detection. The self-capacitance method can be used with the electrode working as a terminal responsible for generating the electric field necessary for capacitance detection. By using a specific receptor for the target UTI marker, these markers can be caught near the touch sensor surface, interacting with the created electric field and, thus, producing a capacitance change, measurable by the touch screen.

These preliminary results, despite short in terms of solution variability and amount of tests, showed great promise. Further work should focus on expanding the tests performed. Urine samples from patients with and without UTI should be tested in order to assess the expected ionic differences between these samples due to presence of nitrite. The touch sensor should also be challenged with *E. coli* solutions for comparison with the first simulation results. Future developments could involve performing detection of these characteristic uropathogens (or “targets”) from this type of infection. Immobilization of these targets by specific “receptors” (as in immunoassays), coated to the CTSS surface, would cause the capacitance change needed to perform capacitive detection (Figure 6). If such technology proves to be viable, a portable biomedical device could be designed to quickly perform UTI detection *in situ*, storing the result and wirelessly sending it to different platforms for analysis.
